# Yoda molecules agonize PIEZO2

**DOI:** 10.64898/2026.05.08.723777

**Published:** 2026-05-12

**Authors:** Tharaka D. Wijerathne, Aneesh Chandrasekharan, Aashish Bhatt, Yun L. Luo, Jérôme J. Lacroix

**Affiliations:** 1 Department of Biomedical Sciences, Western University of Health Sciences, Pomona, CA, USA; 2 Department of Biotechnology and Pharmaceutical Sciences, Western University of Health Sciences, Pomona, CA, USA

**Keywords:** Yoda1, Yoda2, PIEZO2, PIEZO1, mechanotransduction

## Abstract

PIEZO proteins (PIEZO1 and PIEZO2) are essential mechanosensitive channels. PIEZO1 is thought to be selectively activated by Yoda molecules (Yoda1 and Yoda2). Although a structural framework for PIEZO1 activation by Yoda1 exists, a molecular mechanism underlying this selective activation is lacking. Here, using electrophysiology and calcium imaging, we show that Yoda1 increases PIEZO2 open probability and stretch sensitivity as efficaciously as PIEZO1 but elicits weaker PIEZO2-dependent calcium entry, rationalizing why its effect on PIEZO2 has been overlooked. Both Yoda1 and its more potent Yoda2 analog slow down inactivation of PIEZO2 currents with potency similar to PIEZO1 but with lower efficacy. Using mutagenesis and molecular dynamics simulations, we further show that Yoda2’s benzoic acid group forms a transient salt bridge with a conserved arginine in the Yoda binding site, providing a molecular basis for Yoda2’s increased potency. Our study cautions a reevaluation of studies using these molecules to untangle biological functions mediated by PIEZO channels.

## Introduction

PIEZO proteins encompass a small family of mechanically-activated and cation-selective ion channels exclusively present in eukaryotic organisms, with only two vertebrate members called PIEZO1 and PIEZO2 (1, 2). PIEZO channels share the same homotrimeric organization, that of a central ion conducting pore surrounded by three curved blade-like domains, each of these blades consisting of a juxtaposition of nine transmembrane helical bundles (called repeats A-I, or transmembrane helical units 9-1) (3–6). The inherent curvature of the PIEZO blades gives these proteins an overall bowl shape that deforms the surrounding membrane beyond the channel-membrane boundary (7–9). Mechanical membrane deformations such as lateral stretch or outward bending promote flattening of the PIEZO blades (10, 11), a conformational change thought to promote opening of the central pore (12–15).

PIEZO1 is expressed in many cell types, whereas PIEZO2 is mainly present in peripheral mechanosensory neurons as well as a discrete number of non-neuronal cells such as muscle and vascular endothelial cells (2, 16, 17). Genetic studies in which *Piezo1* and/or *Piezo2* genes have been invalidated globally or in specific tissues have revealed numerous biological roles mediated by PIEZO channels, ranging from somatovisceral mechanosensation (18–21) and proprioception (22, 23), to the growth and development of musculo-skeletal (24, 25), cardiac (26), lymphatic (27, 28) and vascular tissues (29, 30). To date, PIEZO channels have been associated with numerous ailments including blood disorders (31–34), mechanical allodynia (35–37), generalized lymphatic dysplasia (38, 39), Prune Belly syndrome (40) and abnormal musculoskeletal development (41–47).

The considerable impact of PIEZO channels on health and diseases has sparked the search for homolog-selective pharmacological modulators, both as molecular probes to dissect homolog-specific biological functions and as potential drugs to treat PIEZO-related pathologies. In this context, Yoda1, a synthetic small molecule proposed to act as a PIEZO1-specific agonist, has been discovered from a high-throughput screening campaign (48). Since its discovery, Yoda1 and its more potent analog Yoda2 (49) have been extensively used as a tool to unravel novel PIEZO1 functions in immune cells (50), bones (51–53), cancer progression (54, 55), and in the vascular system (56–59). In contrast to the common belief that Yoda molecules act as PIEZO1-selective modulators, we show here that they also agonize PIEZO2, prompting careful reinterpretation of prior studies.

## Results

Yoda1 exerts distinct measurable effects when used at micromolar concentrations to PIEZO1-expressing cells: 1) it evokes spontaneous (i.e., without external mechanical stimulation) calcium influx; 2) it reduces the minimal force necessary to detect ionic currents (activation threshold) when these currents are evoked by pipette suction (“stretch currents”) or with cellular indentations (“poking currents”); 3) it slows down inactivation and deactivation kinetics of macroscopic currents; and 4) it increases single channel open probability under steady-state membrane stretch conditions (48, 60, 61). The prevailing notion that Yoda1 acts as a PIEZO1 selective modulator mainly arose from Yoda’s inability to elicit spontaneous calcium influx and to slow down inactivation kinetics of poking currents in PIEZO2-expressing cells (48). The effect of Yoda1 on PIEZO2 stretch currents has never been tested to our knowledge, likely because PIEZO2 was thought to be insensitive to membrane stretch until recently (15, 62, 63).

### Yoda1 robustly modulates PIEZO2 stretch currents

**Figure 1:**
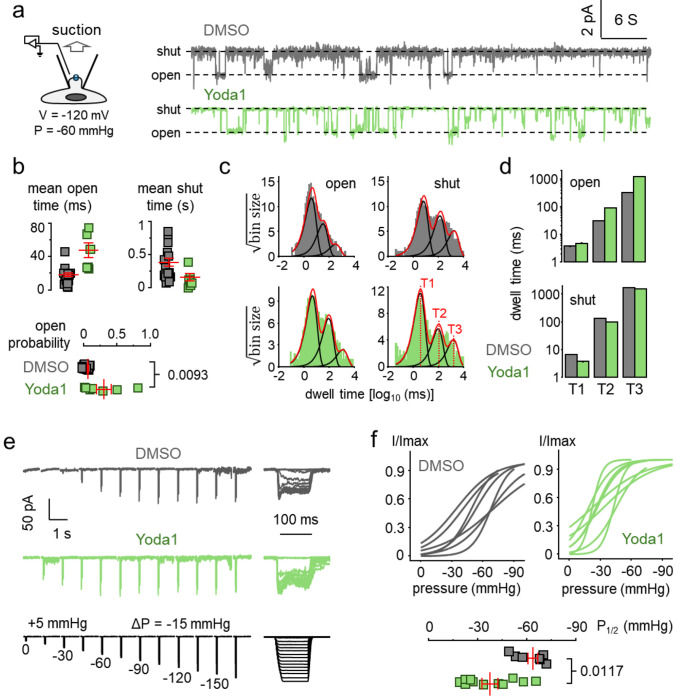
Yoda1 increases PIEZO2’s sensitivity to membrane stretch. **a**) Exemplar PIEZO2 single channel pressure-clamp current trace measured from PIEZO2-transfected HEK293T^ΔPZ1^ cells in presence of 20 μM Yoda1 or 0.05% DMSO vehicle control in the patch pipette. **b**) Scatter plots showing mean open and shut times from idealized single channel current traces (top; DMSO: n = 17, Yoda1: n = 7 independent patches) and open probability calculated for each patch using mean open and shut times (bottom). **c**) Dwell time histograms obtained from single channel recordings (DMSO: 2125 shut/open events; Yoda1: 1457 shut/open events). Histograms were best fitted with three exponential components (red traces) using Maximum likelihood estimation. **d**) Graphs comparing the mean dwell time of each lifetime component. **e**) Exemplar macroscopic PIEZO2 current traces (*left*: concatenated view; *right*: sweep stack view) and evoked by brief suction pulses of varying amplitude in presence of vehicle control or 20 μM Yoda1 in the bath (*Right*: same traces shown as sweep stacks). **f**) *Top*: Graph showing relative PIEZO2 peak current (I/Imax) vs. patch pressure as idealized curves corresponding to Boltzmann fits to the data obtained from independent cells. *Bottom*: Scatter plots of the mid-point activation pressure (P_1/2_) determined from Boltzmann fits shown above (DMSO: n = 9, Yoda1: n = 12 independent cells). Numbers next to bottom plots in (b) and (f) are exact p-values from Mann-Whitney U tests.

We first tested the ability of Yoda1 to modulate PIEZO2 stretch currents. Naïve HEK293T^ΔPZ1^ cells (which do not produce mechanosensitive currents) (38) were transfected with a mouse PIEZO2 plasmid and measured the next day to capture single channel currents in presence of 20 μM Yoda1 or vehicle control (dimethyl sulfoxide, or DMSO) at a constant patch pressure of −60 mmHg (Figure 1a). Idealization of these currents reveal that Yoda1 increases PIEZO2’s mean open time by ~3-fold (18.1 ± 2.4 vs. 47.8 ± 8.3 ms) while decreasing PIEZO2’s mean shut time by ~2-fold (383 ± 62 vs. 161 ± 52 ms). Similar reciprocal microscopic effects of Yoda1 have been reported for PIEZO1 (60). We calculated open probability by dividing the mean open time by the sum of mean open and shut times (64) and found that Yoda1 increases PIEZO2 open probability by about 5-fold in our tested conditions (0.06 ± 0.01 vs. 0.30 ± 0.11, Mann-Whitney U test p-value = 0.0093) (Figure 1b). For comparison, application of 30 μM Yoda1 increases PIEZO1 open probability by 2/3-fold when measured in single channel patches held at −40 mmHg (60). A dwell time analysis of PIEZO2 single channel recordings reveals that the channel populates at least three distinct open and shut states with distinct exponential lifetimes, all of them being reciprocally modulated by Yoda1 (Figure 1c–d), as previously observed for PIEZO1 (60). Next, we measured macroscopic PIEZO2 stretch currents evoked by brief suction pulses of incremental amplitude from +5 to −165 mmHg (Figure 1e). Fitting the relative amplitude of peak currents (I/Imax) using a Boltzmann function indicates that the presence of Yoda1 in the patch pipette correlates with a shift of the mid-point pressure (i.e, pressure needed to reach half of maximal activation, or P_1/2_) by about +20 mmHg (−55.4 ± 5.3 vs. −36 ± 4.5 mmHg, Mann-Whitney U test p-value = 0.0117), similar to the reported +15/20 mmHg shift for PIEZO1’s P_1/2_ value (48, 61). These results show that Yoda1 overall potentiates PIEZO2 stretch currents to a similar extent than reported for PIEZO1 (48, 60).

### Yoda1 activates PIEZO2 in absence of external mechanical stimulus

**Figure 2:**
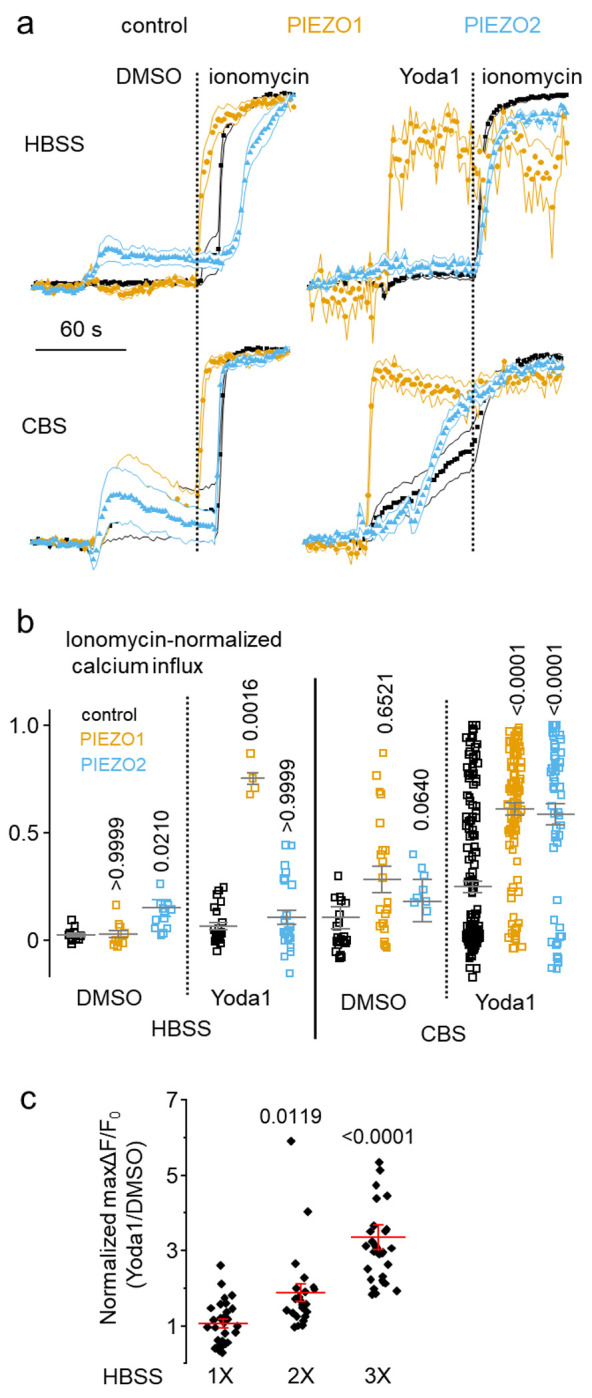
Yoda1 elicits PIEZO2-dependent calcium influx. **a**) Time-course of calcium-sensitive fluorescence obtained with confocal imaging from cells transfected with GC6 (control), with PIEZO1-IRES-GC6 (PIEZO1) or PIEZO2-IRES-GC6 (PIEZO2) plasmids in the presence of Hank’s buffer saline (HBSS) or calcium boosted (CBS) solution. Cells were first exposed to 0.25% DMSO or 30 μM Yoda1 followed by 3 μM ionomycin. Dots and shaded areas respectively represent mean and s.e.m. from n = 12 (control DMSO HBSS), 11 (PIEZO1 DMSO HBSS), 13 (PIEZO2 DMSO HBSS), 23 (control Yoda1 HBSS), 4 (PIEZO1 Yoda1 HBSS), 24 (PIEZO2 Yoda1 HBSS), 26 (control DMSO CBS), 22 (PIEZO1 DMSO CBS), 12 (PIEZO2 DMSO CBS), 183 (control Yoda1 CBS), 105 (PIEZO1 Yoda1 CBS) cells, 55 (PIEZO2 Yoda1 CBS). **b**) Scatter plots comparison of ionomycin-normalized maxΔF/F_0_ obtained from data shown in a). **c**) Calcium influx measured in cells transfected with PIEZO2-IRES-GC6 and elicited by acute perfusion with a HBSS solution at isotonic (1X) or hypertonic (2X and 3X) concentrations containing either 100 μM Yoda1 or DMSO vehicle (Yoda1/DMSO: 1X, n = 29/29; 2X, n = 23/18; 3X, n = 30/30 independent cells). Data are normalized by dividing maxΔF/F_0_ values obtained in each cell treated with Yoda1 by the mean maxΔF/F_0_ value of their corresponding DMSO control. Numbers above plots in b) and c) show p-values from non-parametric Kruskal-Wallis test with Dunn’s multiple comparison against control.

Given the strong modulation of PIEZO2 stretch currents by Yoda1, we sought to re-evaluate the ability of this molecule to elicit spontaneous calcium influx in PIEZO2-expressing cells, i.e, in absence of external mechanical stimulation. Two distinct scenarios could explain why this effect has not been previously observed. First, the ability of Yoda1 to modulate mechanically-evoked PIEZO2 currents (measured electrophysiologically) and to spontaneously activate PIEZO2 (measured using calcium imaging) could be mediated by distinct pharmacological mechanisms, as recently suggested for PIEZO1 (65). On the other hand, the activatory effect of Yoda1 on PIEZO2 might be too weak to be detected in absence of external mechanical stimulus, possibly due to PIEZO2’s lower membrane expression, weaker calcium permeability, and/or inherently higher mechanical activation threshold (63). We thus tested if an effect of Yoda1 can be observed by increasing the amplitude of calcium signals. To this aim, we used an isotonic “calcium boosted solution” (CBS) containing 30 mM Ca^2+^ ions, low potassium and no sodium to increase the inward electrochemical driving force for calcium ions. We transfected naïve cells with a control plasmid encoding the calcium-sensitive fluorescent reporter GCaMP6m (GC6), or with PIEZO1 and PIEZO2 plasmids co-encoding GC6 through an Internal Ribosome Entry Site (IRES) (66). One minute after Yoda1 perfusion, transfected cells were exposed to a calcium ionophore to normalize maximal calcium signals (maxΔF/F_0_) as a function of the maximal GC6 response (to reduce experimental noise due to cell-to-cell variability of plasmid expression). As previously observed, Yoda1 clearly failed to evoke calcium influx through PIEZO2 in the presence of physiological saline (Hank’s Balanced Salt Solution, HBSS) (Figure 2a–b). In contrast, in the presence of CBS, Yoda1 evoked sizeable calcium influx in PIEZO2 transfected cells (Yoda1 vs. DMSO Kruskal-Wallis tests with Dunn’s multiple comparisons p-values <0.0001 for both PIEZO1 and PIEZO2) (Figure 2a–b).

The PIEZO2 structure was recently shown to adopt the characteristic flattened conformation associated with an open state (15) in response to hypertonic, but not hypotonic shocks (67). This seemed counterintuitive since PIEZO1 flattened under hypotonic, but not hypertonic conditions. This conundrum was elegantly explained by the unique property of PIEZO2 to interact with the cytoskeletal-associated protein filamin-B, altering PIEZO2’s response to distinct mechanical stimuli (67). To test if increasing extracellular tonicity can amplify the effect of Yoda1, we compared the amplitude of Yoda1-induced calcium signals (normalized to DMSO vehicle control) in cells transfected with our PIEZO2-IRES-GC6 plasmid under isotonic (1X HBSS) or hypertonic (2X and 3X HBSS) conditions. Calcium signals were approximately 2- to 3-fold larger in hypertonic vs. isotonic conditions (Kruskal-Wallis test with Dunn’s multiple comparisons p-values = 0.0119 and <0.0001 for 2X and 3X, respectively) (Figure 2c).

### Yoda1 modulation of PIEZO2 poking currents

**Figure 3:**
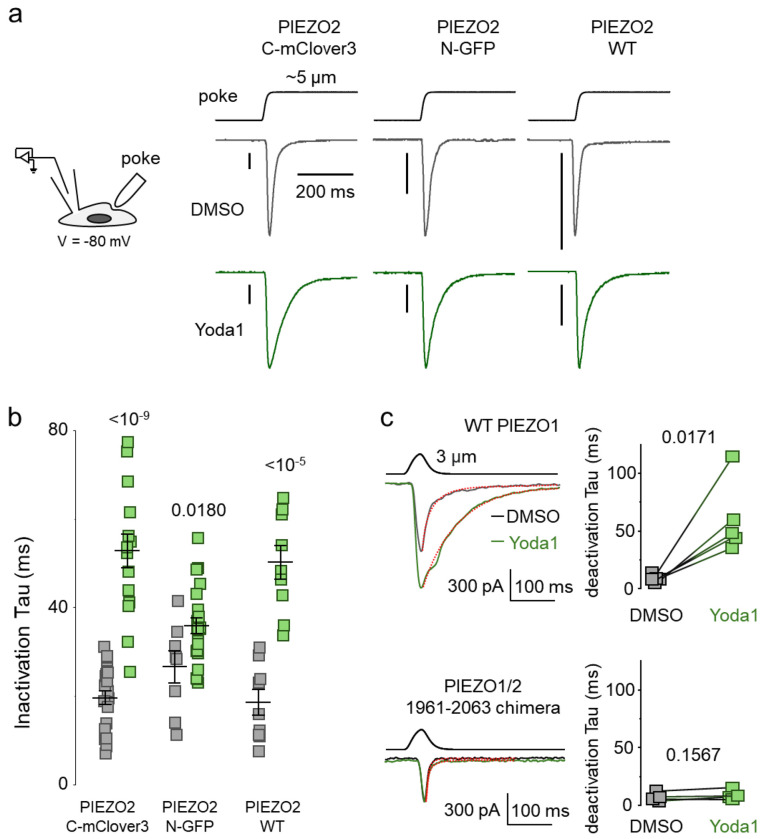
Modulation of PIEZO2 poking currents by Yoda1. **a**) Exemplar poking current traces from cells expressing the indicated PIEZO2 constructs in presence of 30 μM Yoda1 or 0.075% DMSO. Vertical bars represent 200 pA. **b**) Scatter plots showing inactivation Tau obtained from fitting individual traces as shown in a) with a mono-exponential function. Numbers above plots are exact p-values from unpaired t-tests. **c**) *Left*: Whole-cell ionic currents evoked by a 30 ms poke stimulus in HEK293T^ΔPZ1^ cells transfected with a plasmid encoding WT PIEZO1 or the PIEZO1/2 1961-2063 chimera in the presence of 0.075% DMSO (black traces) and after addition of 30 μM Yoda1 (green traces). *Right*: Pairwise comparison of poking current deactivation kinetics (mono-exponential fits are shown as red traces on the left) in the WT (n = 5 independent cells) and chimeric (n = 4 independent cells) channels. Numbers are exact p-values from paired t-tests.

Although Yoda1 was previously found unable to modulate PIEZO2 poking currents kinetics, this conclusion was based on a small sample size (48). We recorded poking currents from a larger number of cells expressing either wild-type mouse PIEZO2 or PIEZO2 variants fused with a green fluorescent protein at the N-terminus (N-GFP) or at the C-terminus (C-mClover3) to better identify cells expressing PIEZO2. As previously observed, PIEZO2 currents display little to no inactivation when elicited by suction pulses (Figure 1e) (63), but exhibit rapid inactivation when elicited by poking stimuli (Figure 3a) (2). Remarkably, PIEZO2 poking currents tend to inactivate about 2-fold slower in the presence of 20 μM Yoda1, an effect that was more pronounced for PIEZO2 C-mClover3 and wild-type (WT) PIEZO2 (unpaired t-test p-value < 10^−5^) than PIEZO2 N-GFP (p-value = 0.0180) (Figure 3b). For comparison, PIEZO1 poking currents inactivate 5-10-fold slower in the presence of Yoda1 in similar experimental conditions (60).

Yoda1 is thought to act as an allosteric positive modulator by binding a hydrophobic pocket sandwiched between PIEZO1 repeats A and B, promoting flattening of the mechanosensory blade domains (61, 65, 68–70). This pharmacological mechanism was initially proposed through engineering a PIEZO1/2 chimera in which a specific amino acid sequence overlapping this binding region (corresponding to mouse PIEZO1 residues 1961-2063) is swapped with its mouse PIEZO2 homologous counterpart (61). This chimera was not modulated by Yoda1 when tested using stretch current protocols, an observation which now seem odd given the present observations that PIEZO2 stretch currents are similarly modulated by Yoda1 (Figure 1e). To complete our electrophysiological characterization, we sought to test an effect of Yoda1 in this chimera with a pairwise poking protocol, using brief poke stimuli to measure deactivation kinetics, the gating parameters being the most modulated by Yoda1 on PIEZO1 (60). Like other tested parameters, deactivation kinetics in this chimera are completely insensitive to 30 μM Yoda1 (p-value =0.1567), in contrast to wild-type PIEZO1 (paired t-test p-value = 0.0171) (Figure 3c).

### Modulation of PIEZO2 poking currents by other small molecules

**Figure 4:**
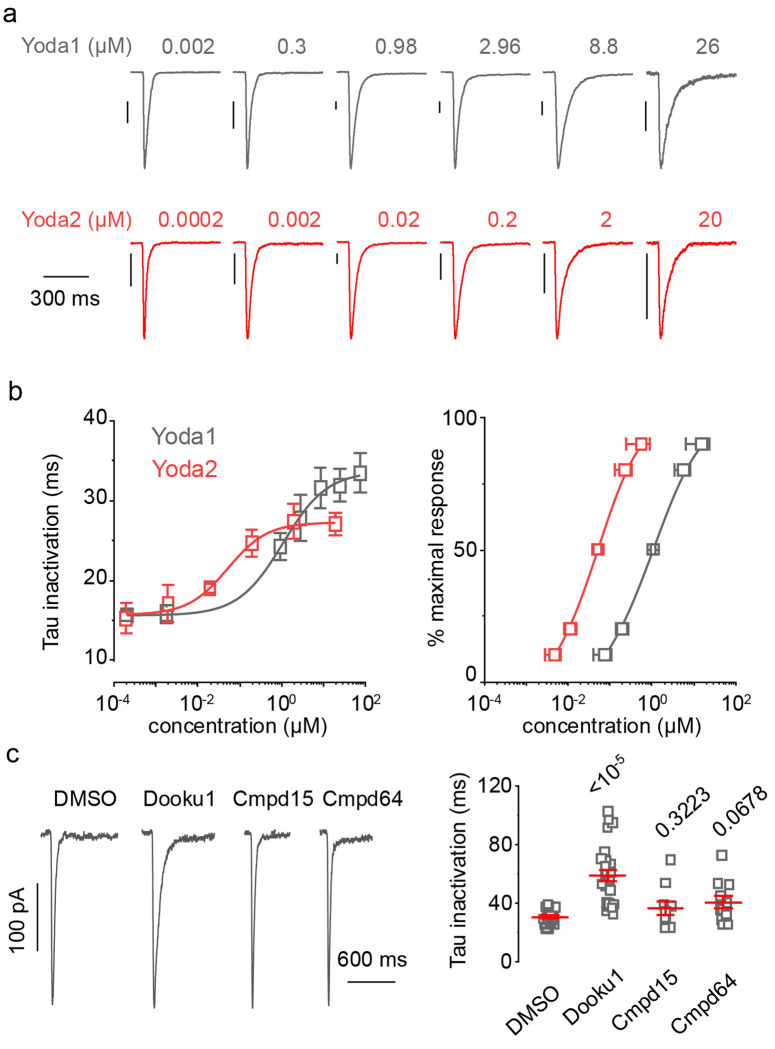
Modulation of PIEZO2 inactivation kinetics by other small molecules. **a**) Exemplar PIEZO2 poking current traces obtained at −80 mV and evoked with a 5 μm poke in the presence of Yoda1 (grey) or Yoda2 (red) in the bath solution at indicated concentrations. Vertical bars represent 200 pA for all traces. **b**) *Left*: Dose-response curves from data shown in (a) depicting mono-exponential Tau inactivation of PIEZO2 poking currents as a function of the concentration of Yoda1 (grey, n = 10, 8, 11, 11, 15, 7, 46 independent cells from low to high dose) or Yoda2 (red, n = 2, 4, 8, 10, 13, 14 46 independent cells from low to high dose) in the bath solution. Lines are fit to the data using a standard dose-response equation. *Right*: Fit-idealized dose-response curves shown on the left are shown here normalized to the maximal response. **c**) *Left*: Exemplar PIEZO2 poking current traces measured in the presence of 30 μM Dooku1, Cmpd15, Cmpd64, or vehicle control (0.1 % DMSO) in the bath solution. *Right*: Scatter plots of Tau inactivation from traces shown in left (DMSO, n = 28; Dooku1, n = 27; Cmpd15, n = 8; Cmpd64, n = 8 independent cells). Numbers above plots show exact p-values from a Kruskal-Wallis test with Dunn’s correction for multiple comparisons.

The replacement of Yoda1’s pyrazine ring for a benzoic acid group led to the creation of Yoda2, an improved analog which agonizes PIEZO1 at nanomolar concentrations (49). We thus sought to test if Yoda2 modulates PIEZO2 currents with similar potency. Although the effect of Yoda1 on PIEZO2 is larger when probed using stretch currents, we used PIEZO2 poking currents instead because these currents are quicker to measure, facilitating the construction of dose-response curves along a wide range of concentrations. Using this approach, we found that Yoda2 slows down PIEZO2 inactivation kinetics at nanomolar concentrations, with a half maximal effective concentration (EC50) at least one order of magnitude lower compared to Yoda1 (~0.4 vs. ~10 μM, Figure 4ab). At saturating concentrations, however, Yoda2 exerts a smaller effect than Yoda1 (lower maximal efficacy). A similar efficacy difference between Yoda1 and Yoda2 has been reported for PIEZO1, as Yoda2 was shown to slow down PIEZO1 poking currents inactivation kinetics by about 1.5-2-fold at saturating concentrations (65), while Yoda1 reportedly slows down inactivation of the same currents by up to 5-10 fold (60).

Next, we tested whether other small molecules can modulate PIEZO2 poking currents, including Dooku1 (a Yoda1 analog that antagonizes the effect of Yoda1 with no known modulatory effect on PIEZO1) and two Yoda1-unrelated PIEZO1 chemical activators (named Cmpd15 and Cmpd64) identified from a structure-guided virtual screening campaign (71, 72). Unexpectedly, Dooku1 slows down inactivation of PIEZO2 poking currents by about 2-3-fold, i.e, similar to Yoda1 (Figure 4c). On the other hand, these currents were not modulated by Cmpds 15 and 64 in our tested conditions, suggesting that these two Yoda-unrelated activators have better PIEZO1-selectivity than Yoda molecules.

### A conserved arginine is required for Yoda2’s nanomolar potency

**Figure 5:**
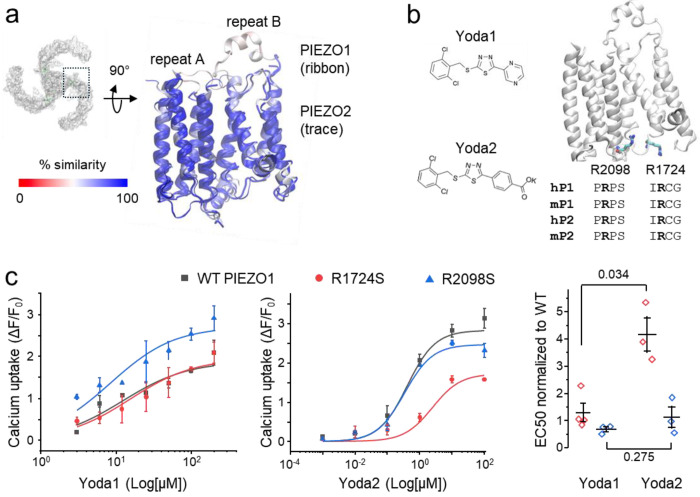
Molecular basis for Yoda2’s increased potency. **a**) Zoomed-in structural alignment of PIEZO1 (PDBID: 6b3R) and PIEZO2 (PDBID: 6kg7) showing the Yoda binding region colored as a function of sequence similarity. **b**) *Left*: chemical structures of Yoda1 and Yoda2. *Right*: localization and sequence conservation of PIEZO1 residues R1724 and R2098 (licorice). **c**) *Left*: dose-responses curves obtained for Yoda1 and Yoda2 against WT PIEZO1 (dark grey), R1724S (red) and R2098S (blue) using calcium imaging measured from n = 3 independent experiments (n = 4 for Yoda1/R1724S). *Right*: scatter plots showing WT-normalized EC50 for both Yoda molecules against each mutant. The numbers next to plots indicate exact p-values from two-tailed Mann-Whitney U-tests.

The Yoda binding region is well conserved between the two channel homologs, both at the level of the backbone structure and of amino acid sequence (Figure 5a) (3–6). It thus seems reasonable to speculate that Yoda molecules modulate PIEZO2 by binding the same region. The intracellular side of this binding region possess two conserved arginine residues, R1724 and R2098 in mouse PIEZO1, which could electrostatically interact with Yoda2’s negatively-charged benzoic acid group, potentially contributing to its higher potency relative to Yoda1 (Figure 5b). Although Yoda1 is electrically neutral, virtual molecular docking studies suggest that mouse PIEZO1 R2098 interacts with Yoda1’s pyrazine group (73–75). We tested a pharmacological role for these basic residues in mouse PIEZO1 by substituting them for serine (to conserve their polar character while eliminating their positive charge) and evaluated the impact of these mutations on Yoda1 and Yoda2 dose-response using calcium imaging (Figure 5c, **left panels**). We fitted these dose-response curves with a standard binding equation and extracted their EC50 values. To ease comparison between mutants and WT channels, we normalized the mutants’ EC50 value to that obtained for WT PIEZO1 and plotted normalized EC50 for both Yoda1 and Yoda2 and for each mutant (Figure 5c, **right panel**). Clearly, the R2098S mutation has no effect on the potency of either Yoda1 or Yoda2, as the normalized EC50 obtained for both molecules is close to unity, ruling out a pharmacological role for R2098. In contrast, the R1724S mutation reduces Yoda2 potency about 4-fold relative to WT (unpaired Student’s t-test p-value = 0.017), with little effect on Yoda1 potency. The large reduction of Yoda2’s potency in the R1724S mutant suggests that R1724 stabilizes Yoda2 by forming a salt bridge.

### Yoda2 forms a transient salt bridge in the Yoda binding site

**Figure 6:**
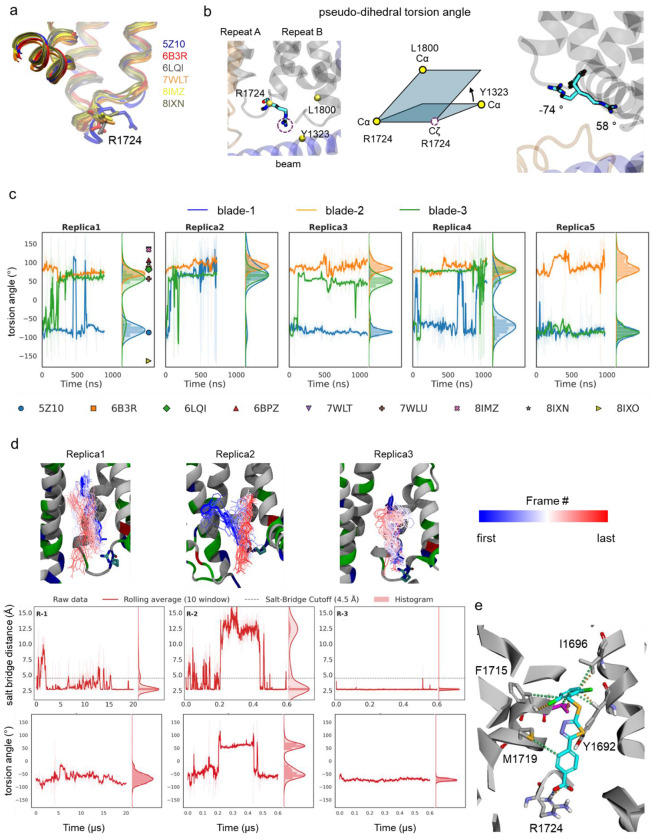
A conserved arginine electrostatically stabilizes Yoda2 in the Yoda binding site. **a**) Structural overlap of indicated PIEZO1 PDBID structures showing rotameric flexibility of R1724. **b**) The side chain orientation of R1724 is calculated by measuring the angle between two planes: one formed by L1800-Cα R1724-Cα and the guanidinium group (R1724-Cζ), the other formed by Y1323-Cα, R1724-Cα and R1724-Cζ. *Right*: example of two R1724 orientations from our simulations with corresponding torsion angle. **c**) Torsion angle dynamics and histogram distribution of R1724 from previous simulations of the full-length PIEZO1 under 9.0 mN/m membrane tension (15). Vertical markers next to the Replica1 histograms indicate torsion angles reported for nine PIEZO1 PDBID structures indicated below. **d**) All-atom simulations of a truncated PIEZO1 blade (3 replicas of 20, 0.6 and 0.6 μs) showing the binding dynamics of Yoda2 (top), the dynamics of Yoda2-R1724 salt-bridge formation (distance between R1724-Cζ and the carboxylic carbon of Yoda2) (middle), and the time-evolution of the R1724 torsion angle (bottom). **e**) The dominant Yoda2 binding pose observed in >55% of the combined simulation frames from 3 replicas shows non-covalent interactions between Yoda2 and the indicated PIEZO1 residues, including the salt bridge with R1724.

The side chain of R1724 displays different orientations in different PIEZO1 experimental structures (Figure 6a). To quantify this orientation, we calculated the pseudo-dihedral torsion angle between the center of the guanidinium group (R1724-Cζ) and three reference atoms (Figure 6b). We next evaluated the temporal distribution of this torsion angle from our recent PIEZO1 simulations (15). Our analysis reveals that this side chain populates two major rotameric orientations, one with torsion angles of 50-100° and the other with torsion angles around −80°. We observed a similar torsion angle distribution across nine PIEZO1 experimental structures (Figure 6c). Critically, rotamers with torsion angles around −80° point the guanidinium group towards the Yoda binding site, while the other rotamers point this group away from it. To explore in more detail how the orientation of R1724 guanidinium group affects Yoda2 binding, we performed all-atom molecular dynamics (MD) simulations of a Yoda2 molecule bound to a truncated PIEZO1 blade (which includes Repeats A-C, the anchor region and the intracellular beam) in the open state, as described in our recent PIEZO1-Yoda1 binding simulations (69) (Figure 6d). Overall, Yoda2 populates binding poses near the intracellular side of the Yoda binding site in the open state, similar to Yoda1 (69). Our analysis reveals a clear correlation between the torsion angle and the formation of a salt-bridge between R1724 and Yoda2: the salt bridge is stable for torsion angle values around −80° and become destabilized when R1724 populates rotamers with positive torsion angles. The dominant Yoda2 binding pose (observed in 82.6%, 49.7%, and 99.8% of all frames in Replica1, 2, and 3, respectively) shows that Yoda2 forms a salt bridge with R1724 and several other non-covalent interactions with I696, F1715, M1719, and Y1692.

## Discussion

Since its discovery, Yoda1 has been used to unravel specific biological functions that depend on PIEZO1 activation. Our discovery that Yoda1 modulates PIEZO2 stretch currents as effectively as PIEZO1’s strongly suggests that some of the reported biological roles attributed to PIEZO1 using this agonist could be (at least partly) transduced by PIEZO2. For instance, our discovery seamlessly reconciles the puzzling fact that reducing PIEZO2 expression in cells that co-express PIEZO1 also reduces the effects of Yoda1 (49, 76, 77). Along the same line, since Yoda1 was shown to increase the expression of the lymphatic valve gene *ITGA9* in a PIEZO1-independent manner (27), this genetic effect could be mediated by PIEZO2, which is present in lymphatic endothelial cells (78). Besides, it is also known that some reported effects of Yoda1 are mediated by proteins unrelated to the PIEZO channel family. For example, Yoda1 was recently shown to inhibit Kv1.3 (79), a major potassium channel in immune cells. Since Yoda1 was shown to modulate certain inflammatory responses, these immunological effects could be mediated independently of PIEZO channel activation, for instance through Kv1.3 inhibition (80–82). Lastly, Yoda1 was shown to induce phosphorylation of protein kinase B (Akt) and extracellular-related kinases (ERK) 1 and 2 in vascular endothelial cells (83) in the presence of GsMTx4, a spider toxin which inhibits mechanosensitive channels including PIEZO1 and 2 (84–86).

We previously engineered a Yoda1-insensitive PIEZO1/2 chimera based on the assumption, now obsolete, that PIEZO2 is insensitive to Yoda1 (61). We however show here that this chimeric swap (swapping mouse PIEZO1 residues 1961-2063 for their homologous mouse PIEZO2 counterparts) completely abolishes Yoda1’s ability to modulates channel kinetics, further strengthening our conclusion that this chimeric PIEZO variant is insensitive to this chemical agonist. A closer look at this chimera reveals that only about half of the residues forming the Yoda1 binding site (those residues contained within repeat A) have been swapped. Although what causes the loss of Yoda1-sensitivity in this chimera remains unclear, we can speculate that the chimeric swap might cause Yoda1 to populate its cognate binding poses less effectively (affinity loss) and/or to adopt novel, non-efficacious binding poses (efficacy loss).

Our pressure-clamp recordings show that Yoda1 lowers PIEZO1’s and PIEZO2’s pressure activation threshold to the same extent. Yet, unlike PIEZO1, a spontaneous activation of PIEZO2 by Yoda1 could only be detected by calcium imaging under non-physiological conditions aiming to increase calcium influx. What molecular mechanisms could explain this difference? A possibility may be due to PIEZO2’s inherently higher pressure-threshold (63, 87). Even in the presence of Yoda1, PIEZO2’s activation threshold would remain higher than the resting membrane tension of the cell, limiting the frequency of spontaneous opening (48, 61, 65). Another possibility stems from the notion that Yoda1 was proposed to adopt two distinct binding poses: an upper pose (near mouse PIEZO1 residues A2091, A2094 and F1693) preferentially occupied by Yoda1 when the curved channel is at rest, and a lower, more intracellular pose (near mouse PIEZO1 residues M1719, F1715, and V1714) populated when the channel flattens (69). Remarkably, the A2094W mutation in the upper site specifically abrogates the activatory, but not modulatory, effects of Yoda1 and also prevents Yoda1 from flattening PIEZO1 in absence of external stimulus (65, 70). This suggests that PIEZO2’s weaker response to Yoda1 in calcium imaging assay could result from alteration of this upper binding site. Such a hypothetical mechanism is likely to be allosteric in nature, since most side chains interacting with Yoda1 are conserved between PIEZO1 and PIEZO2. Another remarkable difference noticed in our study is the lower efficacy of the modulatory effects produced by Yoda molecules on PIEZO2 poking currents inactivation kinetics relative to PIEZO1. This difference may be explained by PIEZO1 and PIEZO2 having distinct interactions with cytoskeletal and adhesion proteins known to modulate inactivation kinetics (67, 88, 89).

Last, our study reveals a role for a conserved arginine located in the Yoda binding site (R1724 in mouse PIEZO1) in selectively increasing the binding potency (lower EC50) of Yoda2. The orientation of this arginine fluctuates during molecular dynamics simulations and across several experimental structures. This rotamer dynamics is likely due to the flexibility of the intracellular loop in which this residue resides. Our binding simulations suggests that, while both Yoda1 and Yoda2 bind with similar poses in the Yoda binding site in the open state, Yoda2’s benzoic acid group forms a salt bridge with this arginine when the latter point its side chain towards the binding site. Hence, we propose that the increased potency of Yoda2 arises from the transient formation of a salt bridge with a flexible arginine side chain. This work thus opens new avenues for drug design and optimization.

## Materials and Methods

### Molecular cloning

We used plasmids encoding mouse PIEZO1 (pCDNA3.1-mPIEZO1) and (PIEZO2Sport6-mPIEZO2) gifted earlier by Ardèm Patapoutian (Scripps Research), as well as a Sport6-mPIEZO2-N-GFP gifted by Tibor Rohacs (Rutgers New Jersey Medical School) and a pMO-mPIEZO2-C-mClover3 plasmid we commissioned Epoch Life Science (TX, USA). The bicistronic plasmids pCDNA3.1-mPIEZO1-IRES-GCaMP6m and pCDNA3.1-mPIEZO2-IRES-GCaMP6m were created in-house earlier (61). mPIEZO1 single point mutations were created and sequence-verified by Epoch Life Science using a pMO-mPIEZO1 plasmid.

### Cell culture, transfection and reagents

HEK293T^ΔPZ1^ cells (gifted earlier from Ardèm Patapoutian), were cultured at 37°C and 5% CO2 in Dulbecco’s Modified Eagle Medium (GIBCO) supplemented with Penicillin-Streptomycin and 5% fetal calf serum (Life technologies). HEK293T^ΔPZ1^ cells were transfected as previously described with slight modifications (90). 3 μg of mPIEZO2 plasmid or 1 μg PIEZO1 plasmid were transfected to 90% confluent HEK293T^ΔPZ1^ cells in a one well of a 12 well plate. Yoda1 (Millipore-Sigma), Yoda2 (Tocris), Dooku1 (Tocris), Cmpd15 (Chembridge chemical ID:7410103) and Cmpd64 (Chembridge chemical ID:33734878) were dissolved as stock solutions in DMSO (Millipore-Sigma) at 40 mM, 40 mM and 10 mM, respectively, as described before (69).

### Electrophysiology

Transfected cells were re-seeded on glass coverslips coated with Matrigel at a 1/50 dilution (Corning) one day after transfection and used for experiments two days after transfection. Patch pipettes were pulled from G150F borosilicate capillaries (Warner Instruments) to a resistance of 2-3 MΩ using a P-97 puller (Sutter Instrument) and heat-polished using a Microforge-MF2 (Narishige). Single channel and macroscopic PIEZO1 and PIEZO2 stretch currents were elicited using gigaseal pressurization (high-speed pressure clamp, ALA Scientific) or by poking cells with a piezo-electric actuator (Physik Instrumente) in a Hank’s Balanced Saline Solution (HBSS, Thermofisher) as described earlier (60). Single channel dwell times were generated and analyzed as described earlier (64).

### Calcium imaging

Cells were washed and incubated for at least 30 min in HBSS prior measurements. The CBS solution contains 1 mM KCl, 30 mM CaCl2, 1 mM MgSO4, 108 mM N-Methyl-D-Glucosamine chloride, and 10 mM HEPES. The pH was adjusted to 7.4 with HCl and has an osmolarity of 288 mOsmol/L (all chemicals were purchased from Millipore-Sigma). For hypertonic treatments, 1X, 2X and 3X HBSS were prepared by dilution of a 10X commercial HBSS solution (Gibco). Fluorescence images were acquired at 1 frame per second either on an inverted Nikon Eclipse microscope through a Nikon CDD camera as described earlier (61), or on a Zeiss LSM880 confocal microscope.

### Molecular dynamics simulations

Docking and molecular dynamics (MD) simulations used a truncated all-atom open PIEZO1 model (residues 1131 to 2190) (69). Molecular docking was performed using AutoDock Vina 1.2.3 with the docking grid defined between Repeat B (TM29–30) and Repeat A (TM35–36) using MGLTools-1.5.6 and a grid size of 30 × 30 × 30 Å. The PIEZO1-Yoda2 complex system was assembled using CHARMM36m parameters for the protein, CHARMM36 parameters for POPC lipids, ions, and water molecules, and CHARMM General Force Field (CGenFF) for Yoda2. The system (211,358 atoms in total) was neutralized and maintained at an ionic concentration of 150 mM KCl and solvated using TIP3P water. All-atom MD simulations were performed using GROMACS 2024.2 smp. We followed the input files generated by CHARMM-GUI, including six equilibration steps followed by production simulations. Energy minimization was performed using the steepest descent algorithm for 5,000 steps. Long-range electrostatic interactions were calculated using the Particle Mesh Ewald (PME) method with a real-space cutoff of 1.2 nm. Van der Waals interactions were smoothly switched off between 1.0 and 1.2 nm using a force-switch function. All hydrogen-containing bonds were constrained using the LINCS algorithm, allowing a 2 fs integration timestep. The system was equilibrated at 310.15 K using the V-rescale thermostat with separate coupling groups for the protein-ligand complex, membrane, and solvent. Semi-isotropic pressure coupling was applied using the C-rescale barostat at 1 bar with a compressibility of 4.5 × 10^−5^ bar^−1^ and a relaxation time constant of 5 ps. Positional restraints were applied during equilibration to the protein backbone, lipids, and dihedral angles.

We obtained three production replicas from three different initial structures. The 20 μs replica was run on Anton3 supercomputer by converting a 100 ns GRO file to a Desmond-compatible format using Viparr on Bridges2. The Anton3 simulation was performed using a multigrator integration scheme with a 2.5 fs timestep. Temperature was maintained using a Nose–Hoover thermostat at 310.15 K and pressure was controlled using the Martyna–Tuckerman–Klein (MTK) barostat with semi-isotropic coupling. Two independent 600 ns replicas were carried out using GROMACS. Ligand–protein interaction analyses were performed using MDAnalysis and PLIP 2.1.0, and molecular visualization was carried out using VMD 1.9.3.

## Data Availability

An experimental data Excel file was deposited into the open-access repository Open Science Framework (DOI: 10.17605/OSF.IO/HQG7D).

## References

[1] Coste B. , Piezo proteins are pore-forming subunits of mechanically activated channels. Nature 483, 176–181 (2012). 22343900 10.1038/nature10812 PMC3297710

[2] Coste B. , Piezo1 and Piezo2 are essential components of distinct mechanically activated cation channels. Science 330, 55–60 (2010). 20813920 10.1126/science.1193270 PMC3062430

[3] Wang L. , Structure and mechanogating of the mammalian tactile channel PIEZO2. Nature 573, 225–229 (2019). 31435011 10.1038/s41586-019-1505-8

[4] Saotome K. , Structure of the mechanically activated ion channel Piezo1. Nature 554, 481–486 (2018). 29261642 10.1038/nature25453 PMC6010196

[5] Zhao Q. , Structure and mechanogating mechanism of the Piezo1 channel. Nature 554, 487–492 (2018). 29469092 10.1038/nature25743

[6] Guo Y. R., MacKinnon R., Structure-based membrane dome mechanism for Piezo mechanosensitivity. Elife 6 (2017). 10.7554/eLife.33660 PMC5788504 29231809

[7] Haselwandter C. A., MacKinnon R., Piezo’s membrane footprint and its contribution to mechanosensitivity. Elife 7 (2018). 10.7554/eLife.41968 PMC6317911 30480546

[8] Haselwandter C. A., Guo Y. R., Fu Z., MacKinnon R., Elastic properties and shape of the Piezo dome underlying its mechanosensory function. Proceedings of the National Academy of Sciences of the United States of America 119, e2208034119 (2022). 36166476 10.1073/pnas.2208034119 PMC9546593

[9] Haselwandter C. A., Guo Y. R., Fu Z., MacKinnon R., Quantitative prediction and measurement of Piezo’s membrane footprint. Proceedings of the National Academy of Sciences of the United States of America 119, e2208027119 (2022). 36166475 10.1073/pnas.2208027119 PMC9546538

[10] Yang X. , Structure deformation and curvature sensing of PIEZO1 in lipid membranes. Nature 10.1038/s41586-022-04574-8 (2022). 35388220

[11] Lin Y. C. , Force-induced conformational changes in PIEZO1. Nature 573, 230–234 (2019). 31435018 10.1038/s41586-019-1499-2 PMC7258172

[12] Jiang W. , Crowding-induced opening of the mechanosensitive Piezo1 channel in silico. Communications Biology 4, 84 (2021). 33469156 10.1038/s42003-020-01600-1 PMC7815867

[13] Liu S. , An intermediate open structure reveals the gating transition of the mechanically activated PIEZO1 channel. Neuron 10.1016/j.neuron.2024.11.020 (2024). 39719701

[14] Ozkan A. D., Wijerathne T. D., Gettas T., Lacroix J. J., Force-induced motions of the PIEZO1 blade probed with fluorimetry. Cell Rep 42, 112837 (2023). 37471225 10.1016/j.celrep.2023.112837 PMC10530446

[15] Li S. , A two-step clockwork mechanism opens a proteo-lipidic pore in PIEZO2. Nat. Chem. Biol. 10.1038/s41589-026-02147-8 (2026). PMC13423835 41703062

[16] David L. , Piezo mechanosensory channels regulate centrosome integrity and mitotic entry. Proc. Natl. Acad. Sci. U. S. A. 120, e2213846120 (2023). 36574677 10.1073/pnas.2213846120 PMC9910506

[17] Wei F. , Deficiency of Endothelial Piezo2 Impairs Pulmonary Vascular Angiogenesis and Predisposes Pulmonary Hypertension. Hypertension 82, 583–597 (2025). 39758000 10.1161/HYPERTENSIONAHA.124.22948

[18] Ranade S. S. , Piezo2 is the major transducer of mechanical forces for touch sensation in mice. Nature 516, 121–125 (2014). 25471886 10.1038/nature13980 PMC4380172

[19] Woo S. H. , Piezo2 is required for Merkel-cell mechanotransduction. Nature 509, 622–626 (2014). 24717433 10.1038/nature13251 PMC4039622

[20] Ehlers V. L., Sriram A., Stuart B. A. R., Mecca C. M., Stucky C. L., Sensory neuron PIEZO1 deletion inhibits dynamic light touch sensitivity in uninjured mice, prevents neuropathic light touch hypersensitivity, and drives compensatory changes in dorsal root ganglia. Pain 10.1097/j.pain.0000000000003781 (2025). PMC12598664 40892990

[21] Servin-Vences M. R. , PIEZO2 in somatosensory neurons controls gastrointestinal transit. Cell 186, 3386–3399 e3315 (2023). 37541196 10.1016/j.cell.2023.07.006 PMC10501318

[22] Florez-Paz D., Bali K. K., Kuner R., Gomis A., A critical role for Piezo2 channels in the mechanotransduction of mouse proprioceptive neurons. Scientific reports 6, 25923 (2016). 27184818 10.1038/srep25923 PMC4869095

[23] Woo S. H. , Piezo2 is the principal mechanotransduction channel for proprioception. Nature neuroscience 18, 1756–1762 (2015). 26551544 10.1038/nn.4162 PMC4661126

[24] Sun W. , The mechanosensitive Piezo1 channel is required for bone formation. Elife 8 (2019). 10.7554/eLife.47454 PMC6685704 31290742

[25] Li X. , Stimulation of Piezo1 by mechanical signals promotes bone anabolism. Elife 8 (2019). 10.7554/eLife.49631 PMC6779475 31588901

[26] Jiang F. , The mechanosensitive Piezo1 channel mediates heart mechano-chemo transduction. Nat Commun 12, 869 (2021). 33558521 10.1038/s41467-021-21178-4 PMC7870949

[27] Choi D. , Piezo1 incorporates mechanical force signals into the genetic program that governs lymphatic valve development and maintenance. JCI Insight 4, 125068 (2019). 30676326 10.1172/jci.insight.125068 PMC6483520

[28] Nonomura K. , Mechanically activated ion channel PIEZO1 is required for lymphatic valve formation. Proc. Natl. Acad. Sci. U. S. A. 115, 12817–12822 (2018). 30482854 10.1073/pnas.1817070115 PMC6294938

[29] Li J. , Piezo1 integration of vascular architecture with physiological force. Nature 515, 279–282 (2014). 25119035 10.1038/nature13701 PMC4230887

[30] Ranade S. S. , Piezo1, a mechanically activated ion channel, is required for vascular development in mice. Proceedings of the National Academy of Sciences of the United States of America 111, 10347–10352 (2014). 24958852 10.1073/pnas.1409233111 PMC4104881

[31] Cahalan S. M. , Piezo1 links mechanical forces to red blood cell volume. Elife 4 (2015). 10.7554/eLife.07370 PMC4456639 26001274

[32] Demolombe S., Duprat F., Honore E., Patel A., Slower Piezo1 inactivation in dehydrated hereditary stomatocytosis (xerocytosis). Biophys. J. 105, 833–834 (2013). 23972832 10.1016/j.bpj.2013.07.018 PMC3752110

[33] Bae C., Gnanasambandam R., Nicolai C., Sachs F., Gottlieb P. A., Xerocytosis is caused by mutations that alter the kinetics of the mechanosensitive channel PIEZO1. Proc. Natl. Acad. Sci. U. S. A. 110, E1162–1168 (2013). 23487776 10.1073/pnas.1219777110 PMC3606986

[34] Romero L. O. , Enhanced PIEZO1 function contributes to the pathogenesis of sickle cell disease. Proc. Natl. Acad. Sci. U. S. A. 122, e2514863122 (2025). 41037639 10.1073/pnas.2514863122 PMC12519228

[35] Szczot M. , PIEZO2 mediates injury-induced tactile pain in mice and humans. Sci Transl Med 10 (2018). 10.1126/scitranslmed.aat9892 PMC6875774 30305456

[36] Murthy S. E. , The mechanosensitive ion channel Piezo2 mediates sensitivity to mechanical pain in mice. Sci Transl Med 10 (2018). 10.1126/scitranslmed.aat9897 PMC6709986 30305457

[37] Eijkelkamp N. , A role for Piezo2 in EPAC1-dependent mechanical allodynia. Nat Commun 4, 1682 (2013). 23575686 10.1038/ncomms2673 PMC3644070

[38] Lukacs V. , Impaired PIEZO1 function in patients with a novel autosomal recessive congenital lymphatic dysplasia. Nature communications 6, 8329 (2015). 10.1038/ncomms9329 PMC4578306 26387913

[39] Fotiou E. , Novel mutations in PIEZO1 cause an autosomal recessive generalized lymphatic dysplasia with non-immune hydrops fetalis. Nat Commun 6, 8085 (2015). 26333996 10.1038/ncomms9085 PMC4568316

[40] Amado N. G. , PIEZO1 loss-of-function compound heterozygous mutations in the rare congenital human disorder Prune Belly Syndrome. Nat Commun 15, 339 (2024). 38184690 10.1038/s41467-023-44594-0 PMC10771463

[41] Ma S. , Excessive mechanotransduction in sensory neurons causes joint contractures. Science 379, 201–206 (2023). 36634173 10.1126/science.add3598 PMC10163824

[42] Haliloglu G. , Recessive PIEZO2 stop mutation causes distal arthrogryposis with distal muscle weakness, scoliosis and proprioception defects. J. Hum. Genet. 62, 497–501 (2017). 27974811 10.1038/jhg.2016.153

[43] Alisch F. , Familial Gordon syndrome associated with a PIEZO2 mutation. Am J Med Genet A 10.1002/ajmg.a.37997 (2016). 27714920

[44] Mahmud A. A. , Loss of the proprioception and touch sensation channel PIEZO2 in siblings with a progressive form of contractures. Clin. Genet. 10.1111/cge.12850 (2016). 27607563

[45] Okubo M. , A family of distal arthrogryposis type 5 due to a novel PIEZO2 mutation. Am J Med Genet A 167A, 1100–1106 (2015). 25712306 10.1002/ajmg.a.36881

[46] McMillin M. J. , Mutations in PIEZO2 cause Gordon syndrome, Marden-Walker syndrome, and distal arthrogryposis type 5. Am. J. Hum. Genet. 94, 734–744 (2014). 24726473 10.1016/j.ajhg.2014.03.015 PMC4067551

[47] Coste B. , Gain-of-function mutations in the mechanically activated ion channel PIEZO2 cause a subtype of Distal Arthrogryposis. Proc. Natl. Acad. Sci. U. S. A. 110, 4667–4672 (2013). 23487782 10.1073/pnas.1221400110 PMC3607045

[48] Syeda R. , Chemical activation of the mechanotransduction channel Piezo1. Elife 4 (2015). 10.7554/eLife.07369 PMC4456433 26001275

[49] Parsonage G. , Improved PIEZO1 agonism through 4-benzoic acid modification of Yoda1. Br. J. Pharmacol. 10.1111/bph.15996 (2022). PMC10952572 36457143

[50] Jäntti H. , Microglial amyloid beta clearance is driven by PIEZO1 channels. J. Neuroinflammation 19 (2022). 10.1186/s12974-022-02486-y PMC9199162 35706029

[51] Shindo S. , Piezo1 protects against inflammatory bone loss via a unique Ca-independent mechanism in osteoclasts. Front. Immunol. 16 (2025). 10.3389/fimmu.2025.1661538 PMC12507952 41080556

[52] He X. , Yoda1 pretreated BMSC derived exosomes accelerate osteogenesis by activating phospho-ErK signaling via Yoda1-mediated signal transmission. J Nanobiotechnol 22 (2024). 10.1186/s12951-024-02669-0 PMC11234696 38987801

[53] Meslier Q. A., Oehrlein R., Shefelbine S. J., Combined Effects of Mechanical Loading and Piezo1 Chemical Activation on 22-Months-Old Female Mouse Bone Adaptation. Aging Cell 24, e70087 (2025). 40410950 10.1111/acel.70087 PMC12341773

[54] Silvani G. , Capillary constrictions prime cancer cell tumorigenicity through PIEZO1. Nature Communications 16 (2025). 10.1038/s41467-025-63374-6 PMC12402161 40890143

[55] Liao W. X. , The activation of Piezo1 channel promotes invasion and migration via the release of extracellular ATP in cervical cancer. Pathology Research and Practice 260 (2024). 10.1016/j.prp.2024.155426 38908334

[56] Jung J. U., Stippec S., Cobb M. H., Activation of WNK1 signaling through Piezo1. Proc. Natl. Acad. Sci. U. S. A. 122 (2025). 10.1073/pnas.2513155122 PMC12415193 40880539

[57] Lone M. , Activation of the Piezo1 channel stimulates protein kinase D and migration in human aortic endothelial cells. Am. J. Physiol. Cell Physiol. 329, C1652–C1665 (2025). 41071645 10.1152/ajpcell.00457.2025 PMC12782040

[58] Endesh N. , Independent endothelial functions of PIEZO1 and TRPV4 in hepatic portal vein and predominance of PIEZO1 in mechanical and osmotic stress. Liver Int 43, 2026–2038 (2023). 37349903 10.1111/liv.15646 PMC10946873

[59] Qiao M. , Piezo1 Channel Activators Yoda1 and Yoda2 in the Context of Red Blood Cells. Biomolecules 15 (2025). 10.3390/biom15081110 PMC12384225 40867555

[60] Wijerathne T. D., Ozkan A. D., Lacroix J. J., Yoda1’s energetic footprint on Piezo1 channels and its modulation by voltage and temperature. Proceedings of the National Academy of Sciences of the United States of America 10.1073/pnas.2202269119, e2202269119 (2022). 35858335 PMC9303978

[61] Lacroix J. J., Botello-Smith W. M., Luo Y., Probing the gating mechanism of the mechanosensitive channel Piezo1 with the small molecule Yoda1. Nature Communications 9, 2029 (2018). 10.1038/s41467-018-04405-3 PMC5966384 29795280

[62] Shin K. C. , The Piezo2 ion channel is mechanically activated by low-threshold positive pressure. Sci. Rep. 9, 6446 (2019). 31015490 10.1038/s41598-019-42492-4 PMC6478859

[63] Murthy S. E., Deciphering mechanically activated ion channels at the single-channel level in dorsal root ganglion neurons. J Gen Physiol 155 (2023). 10.1085/jgp.202213099 PMC10140383 37102984

[64] Wijerathne T. D., Ozkan A. D., Lacroix J. J., Microscopic mechanism of PIEZO1 activation by pressure-induced membrane stretch. J. Gen. Physiol. 155 (2023). 10.1085/jgp.202213260 PMC9930135 36715688

[65] Verkest C., Roettger L., Zeitzschel N., Lechner S. G., 3D-MINFLUX nanoscopy reveals distinct allosteric mechanisms for activation and modulation of PIEZO1 by Yoda1. Nat Commun 16, 11192 (2025). 41402296 10.1038/s41467-025-67610-x PMC12712055

[66] Chen T. W. , Ultrasensitive fluorescent proteins for imaging neuronal activity. Nature 499, 295–300 (2013). 23868258 10.1038/nature12354 PMC3777791

[67] Mulhall E. M., Yarishkin O., Hill R. Z., Koster A. K., Patapoutian A., The molecular basis of force selectivity by PIEZO2. Nature 10.1038/s41586-026-10182-7 (2026). PMC13149025 41781615

[68] Wang Y. , A lever-like transduction pathway for long-distance chemical- and mechano-gating of the mechanosensitive Piezo1 channel. Nat Commun 9, 1300 (2018). 29610524 10.1038/s41467-018-03570-9 PMC5880808

[69] Jiang W. , Structural and thermodynamic framework for PIEZO1 modulation by small molecules. Proc. Natl. Acad. Sci. U. S. A. 120, e2310933120 (2023). 38060566 10.1073/pnas.2310933120 PMC10723123

[70] Botello-Smith W. M. , A mechanism for the activation of the mechanosensitive Piezo1 channel by the small molecule Yoda1. Nature Communications 10, 4503 (2019). 10.1038/s41467-019-12501-1 PMC6776524 31582801

[71] Wijerathne T. D., Ozkan A. D., Lacroix J. J., Thermodynamic and structural bases for Yoda1-mediated Piezo1 activation. Biophys. J. 121, 176a (2022).

[72] Evans E. L. , Yoda1 analogue (Dooku1) which antagonizes Yoda1-evoked activation of Piezo1 and aortic relaxation. Br. J. Pharmacol. 175, 1744–1759 (2018). 29498036 10.1111/bph.14188 PMC5913400

[73] Goon S. , Exploring the Structural Attributes of Yoda1 for the Development of New-Generation Piezo1 Agonist Yaddle1 as a Vaccine Adjuvant Targeting Optimal T Cell Activation. J. Med. Chem. 67, 8225–8246 (2024). 38716967 10.1021/acs.jmedchem.4c00322

[74] Tang H. , Structural Modification and Pharmacological Evaluation of (Thiadiazol-2-yl)pyrazines as Novel Piezo1 Agonists for the Intervention of Disuse Osteoporosis. J. Med. Chem. 67, 19837–19851 (2024). 39462841 10.1021/acs.jmedchem.4c02224

[75] Xu K., Liu M., Wang L., Zhan G., He G., Piezo Family: From Structure to Therapeutic Agents in Cancer. J. Med. Chem. 10.1021/acs.jmedchem.5c03200 (2026). 41723711

[76] Dalghi M. G. , Functional roles for PIEZO1 and PIEZO2 in urothelial mechanotransduction and lower urinary tract interoception. JCI Insight 6 (2021). 10.1172/jci.insight.152984 PMC8525643 34464353

[77] Savadipour A. , Membrane stretch as the mechanism of activation of PIEZO1 ion channels in chondrocytes. Proc. Natl. Acad. Sci. U. S. A. 120, e2221958120 (2023). 37459546 10.1073/pnas.2221958120 PMC10372640

[78] Akita S. , Increased expression of the PIEZO2 mechanoreceptor in fibroblasts and endothelial cells within the lymphatic and vascular vessels of keloids. J. Pathol. 267, 105–119 (2025). 40742741 10.1002/path.6455 PMC12337814

[79] Nguyen H. M., Huang K. Y., Cui Y. J., Shim H., Wulff H., Off-target effects of Piezo1 agonists on macrophages. Biophys. J. 124 (2025).

[80] Pourteymour S. , PIEZO1 targeting in macrophages boosts phagocytic activity and foam cell apoptosis in atherosclerosis. Cell Mol Life Sci 81, 331 (2024). 39107572 10.1007/s00018-024-05372-3 PMC11335255

[81] Xiao Y. , ZnCe-LDO Nanozyme-Based Multifunctional Hydrogel Promotes Bone Regeneration by Inflammatory Macrophage Reprogramming and Piezo1 Activation. ACS Nano 19, 32606–32628 (2025). 40911285 10.1021/acsnano.5c10123 PMC12445009

[82] Shindo S. , Piezo1 protects against inflammatory bone loss via a unique Ca(2+)-independent mechanism in osteoclasts. Front. Immunol. 16, 1661538 (2025). 41080556 10.3389/fimmu.2025.1661538 PMC12507952

[83] dela Paz N. G., Frangos J. A., Yoda1-induced phosphorylation of Akt and ERK1/2 does not require Piezol activation. Biochemical and Biophysical Research Communications 497, 220–225 (2018). 29428723 10.1016/j.bbrc.2018.02.058 PMC5835220

[84] Alcaino C., Knutson K., Gottlieb P. A., Farrugia G., Beyder A., Mechanosensitive ion channel Piezo2 is inhibited by D-GsMTx4. Channels (Austin) 11, 245–253 (2017). 28085630 10.1080/19336950.2017.1279370 PMC5463886

[85] Bae C., Sachs F., Gottlieb P. A., The mechanosensitive ion channel Piezo1 is inhibited by the peptide GsMTx4. Biochemistry 50, 6295–6300 (2011). 21696149 10.1021/bi200770q PMC3169095

[86] Ostrow K. L. , cDNA sequence and in vitro folding of GsMTx4, a specific peptide inhibitor of mechanosensitive channels. Toxicon 42, 263–274 (2003). 14559077 10.1016/s0041-0101(03)00141-7

[87] Lewis A. H., Grandl J., Mechanical sensitivity of Piezo1 ion channels can be tuned by cellular membrane tension. Elife 4 (2015). 10.7554/eLife.12088 PMC4718726 26646186

[88] Wang J. , Tethering Piezo channels to the actin cytoskeleton for mechanogating via the cadherin-beta-catenin mechanotransduction complex. Cell Rep 38, 110342 (2022). 35139384 10.1016/j.celrep.2022.110342

[89] Koster A. K. , Chemical mapping of the surface interactome of PIEZO1 identifies CADM1 as a modulator of channel inactivation. Proc. Natl. Acad. Sci. U. S. A. 121, e2415934121 (2024). 39356664 10.1073/pnas.2415934121 PMC11474052

[90] Wijerathne T. D., Bhatt A., Jiang W., Luo Y. L., Lacroix J. J., Mammalian PIEZO channels rectify anionic currents. Biophys J 10.1016/j.bpj.2024.11.010 (2024). PMC12820994 39543876

